# Bridging the biostatistics gap in African health research: An urgent call to action

**DOI:** 10.4314/ahs.v25i3.14

**Published:** 2025-09

**Authors:** Michal Ordak

**Affiliations:** Department of Pharmacotherapy and Pharmaceutical Care, Faculty of Pharmacy, Medical University of Warsaw, Banacha 1 Str., 02-097 Warsaw, Poland

**Keywords:** Biostatistics, African health research

## Abstract

The shortage of biostatisticians across Africa continues to hinder the quality, transparency, and reproducibility of health research on the continent. Drawing on documented regional initiatives and published literature, this Letter to the Editor highlights the ongoing gap in biostatistical capacity and proposes three practical recommendations to address it. These include the organization of structured training through pan-African institutions, the promotion of existing statistical reporting guidelines such as SAMPL, and the integration of applied biostatistics into doctoral and medical education. Strengthening statistical competence through these scalable approaches could support researchers, improve the quality of published research, and foster more rigorous scientific practice throughout Africa.

Over the past decade and a half, multiple initiatives and discussions have attempted to address a critical weakness in health research capacity across Africa: the persistent shortage of trained biostatisticians. This issue, though widely acknowledged, continues to hinder the quality and reproducibility of scientific findings emanating from the continent. As early as 2011, a workshop organized by the U.S. National Institutes of Health (NIH) highlighted the growing investment in biomedical research across sub-Saharan Africa (SSA), largely driven by the HIV/AIDS epidemic, reemerging infectious diseases, and increased international engagement in global health. Yet, alongside these developments, a key concern emerged—the need to bolster local biostatistics expertise to ensure that research data could be properly analyzed and interpreted. Discussions emphasized not only the demand for skilled biostatisticians to participate in ongoing projects, but also the importance of building a sustainable infrastructure that could educate and train future generations of African biostatistical researchers^1^. In 2015, a focused workshop involving African biostatisticians and methodologists again drew attention to the scarcity of biostatistics professionals across SSA. This meeting acknowledged that previous recommendations for strengthening biostatistics capacity had not been implemented. As a response, participants proposed the establishment of the Africa Center for Biostatistical Excellence - a regional, collaborative initiative aimed at advancing postgraduate training and regular workshops to address this systemic skill gap^2^. By 2020, some progress had been made. The Sub-Saharan African Consortium for Advanced Biostatistics (SSACAB) was launched as a major capacity-building program led by African universities, with support from European partners. SSACAB played a crucial role in expanding the number of master's programs in biostatistics from just four in 2015 to eleven by 2020. It awarded over 150 fellowships and facilitated the publication of dozens of peer-reviewed articles. While commendable, these efforts remain limited in scope compared to the magnitude of need across the continent^3^. More recently, in 2023, the Vanderbilt-Nigeria Biostatistics Training Program (VN-BioStat) was established to build biostatistical leadership in Nigeria, focusing on HIV research. Through structured fellowships, international mentorship, and research-based training, this initiative reflects a growing recognition of the strategic role that biostatistics plays in shaping health outcomes and research quality. However, the program, while valuable, serves only a narrow geographic and disciplinary niche, further underscoring the need for continent-wide solutions^4^. Taken together, these efforts reflect increasing awareness but also a pattern of fragmented and under-scaled responses. Despite years of discussion, there remains no unified or sustained strategy to build biostatistics capacity across Africa in a way that is accessible, coordinated, and tailored to the continent's diverse research environments. In this context, drawing on my experience as a statistical editor and reviewer for multiple scientific journals, I wish to propose a set of scalable, collaborative, and practical solutions to support biostatistics development across Africa.

First, I recommend that African research institutions, universities, and public health agencies explore partnerships with established pan-African institutions such as the African Academy of Sciences (AAS) to deliver structured biostatistics training. The Academy's broad network of researchers, fellows, and affiliated institutions positions it well to support continent-wide efforts to improve statistical competencies in health research. Online workshops hosted under the auspices of such organizations could provide African researchers with critical skills in statistical analysis, including appropriate test selection, assumption checking, effect size reporting, and proper interpretation of results. These sessions could be incorporated into existing AAS events or offered as standalone programs targeting early- and mid-career researchers. Furthermore, such training could be embedded within major AAS-supported scientific initiatives - such as those focused on malaria or emerging infectious diseases - ensuring that statistical rigor is integrated from the design phase onward.

Second, I recommend encouraging the wider use of existing statistical reporting guidelines such as the Statistical Analyses and Methods in the Published Literature (SAMPL) guidelines^5^ as a practical way to strengthen the statistical quality of African research publications. Although these recommendations have been available since 2015, they are still not widely adopted. A recent publication from March 2025 emphasized that broader implementation of the SAMPL Guidelines can help improve transparency, clarity, and methodological rigor in biomedical research. Key areas such as articulating the purpose of statistical tests, verifying assumptions, reporting effect sizes, and addressing outliers are all strengthened when these guidelines are applied^6^. Promoting their use in research training programs and manuscript development resources could support African scientists in producing more robust and interpretable results. The SAMPL Guidelines are available on the website of the World Association of Medical Editors (WAME) and may serve as a practical reference for both authors and editorial teams.

Third, I recommend integrating biostatistics more meaningfully into doctoral and medical training programs within African academic institutions, as a long-term strategy to improve research quality and statistical literacy. Many of the reporting issues seen in submitted manuscripts likely stem not from intentional misuse but from limited access to structured and relevant biostatistics education. One recent study among medical students in Europe found that teaching approaches based on real-life articles and practical applications significantly reduced students' stress and improved their learning outcomes, compared to traditional lecture-based instruction^7^. While the context differs, the principle of grounding statistical education in meaningful, applied examples could be adapted to medical and graduate programs across Africa. Similarly, analyses of PhD-level training in biomedical sciences have recommended incorporating tailored, field-specific statistical instruction as a core part of research education^8^. Encouraging African universities and public health training institutions to adopt these evidence-informed teaching practices could help prepare future researchers to approach statistical analysis with greater confidence, improving the quality of study design, data interpretation, and ultimately, published research.

These recommendations are presented in support of ongoing efforts to enhance the quality of health research across Africa, particularly through improved statistical capacity. Consideration of these perspectives may contribute to future strategies aimed at strengthening methodological rigor in scientific publishing.
